# Five challenging presentations of juvenile nasopharyngeal angiofibroma: our experience in the diagnosis and endoscopic surgical management

**DOI:** 10.1093/jscr/rjaf055

**Published:** 2025-02-28

**Authors:** Danah Alrusayyis, Jood K Alotaibi, Rahaf Almutarii, Fadhel M Almolani, Mohammed Al-Ahmari, Abdulrahman Alkhatib, Ali Almomen

**Affiliations:** Department of Otolaryngology, Eastern Health Cluster, Dammam, Saudi Arabia; College of Medicine, Imam Abdulrahman bin Faisal University, Dammam, Saudi Arabia; College of Medicine, Imam Mohammad bin Saud University, Riyadh, Saudi Arabia; Department of Radiology, King Fahad Specialist Hospital, Dammam, Saudi Arabia; Department of Otolaryngology-Head and Neck Surgery, King Fahad Specialist Hospital, Dammam, Saudi Arabia; Department of Otolaryngology-Head and Neck Surgery, King Fahad Specialist Hospital, Dammam, Saudi Arabia; Department of Otolaryngology-Head and Neck Surgery, King Fahad Specialist Hospital, Dammam, Saudi Arabia

**Keywords:** juvenile nasopharyngeal angiofibroma, case series, recurrence

## Abstract

Juvenile nasopharyngeal angiofibroma (JNA) is a benign tumor originating from the pterygopalatine fossa and is the most common lesion of the nasopharynx. Its hypervascularity and complex anatomical extensions make selecting the most effective surgical approach intricate. All patients in this series were males, and their age ranged between 8 and 23 years. Extensions to the carotids, cavernous sinus, and infratemporal fossa were noted. In addition, intraoperative details with particular emphasis on the endoscopic two-surgeon transseptal approach were discussed. Nasal obstruction was the most reported symptom, whereas epistaxis was evident in two cases. Complete or near-complete resection of the tumor was achieved in all cases. Over the follow-up period, one patient underwent successful revision endoscopic excision after tumor regrowth. JNA can be completely excised with endoscopic techniques. For large tumors, creating a wider window by using a two-surgeon trans-septal approach is practical and defers the need for external resection.

## Introduction

Juvenile nasopharyngeal angiofibroma (JNA) is a pseudoencapsulated, hypervascular tumor that typically presents with mild symptoms, including nasal obstruction, facial numbness, and spontaneous epistaxis [1–3]. Although JNA accounts for only 0.05%–0.5% of head and neck tumors, its clinical significance lies in its locally aggressive behavior [4]. Despite its benign histopathology, JNA spreads in a submucosal and subperioseal fashion to adjacent structures, such as cranial nerves, major vessels, and the cavernous sinus [3, 5–8]. The hypervascularity and complex anatomical extension of the tumor make choosing the surgical approach intricate. While there is a consensus that tumors confined to the nasopharynx, pterygopalatine fossa, and paranasal sinuses can be resected endoscopically, the choice between external and endoscopic surgery to resect tumors extending to the infratemporal fossa or skull base remains contentious [3, 8–10].

This paper presents our tailored management approach for five cases of JNA, each characterized by unique challenges. These include large tumor size, extensive bilateral blood supply, lateral infratemporal fossa extension, proximity to the carotid artery, critical extension to the internal carotid artery (ICA), and complications related to embolization.

## Case presentations

This case series includes five male patients, with ages at presentation ranging from 9 to 23 years. Details about the patient characteristics and management are outlined in Table 1. All patients presented with nasal obstruction, while only three experienced epistaxis. Histopathologic Examination of the mass confirmed the diagnosis of JNA, and coagulation disorders were ruled out. A total of six endoscopic resection procedures, along with arterial embolization were performed.

**Table 1:** Characteristics of included patients and their management.

**No.**	**Age**	**Symptoms**	**Primary site**	**Involved sinuses**	**Size (cm)**	**Embolization**	**Complications**	**Surveillance**
1	9	Nasal obstruction, epistaxis	Left nasal cavity and nasopharynx	Left maxillary and ethmoid, bilateral sphenoid	6.9 × 5.9 × 5.0	Left and right ECA	None	Recurrence after 8 months
2	23	Nasal obstruction, epistaxis, headache	Left pterygopalatine fossa	Left sphenoid	5.1 × 4.5 × 4.7	Left IMA	None	12 years: disease-free
3	13	Nasal obstruction, epistaxis	Left pterygopalatine fossa	None	4.0 × 2.5 × 1.0	Left IMA	None	18 months; disease-free
4	13	Nasal obstruction, mouth breathing, snoring, ear blockage	Left intranasal	Sphenoid and maxillary		Left ECA	Otalgia, tinnitus, headache, ↓ visual acuity	No recurrence after revision surgery
5	12	Nasal obstruction, snoring	Right intranasal	Sphenoid and ethmoid		Right IMA, right MMA, and right ICA	Post-embolization brain infarction	Recurrence after 6 months

ECA: External Carotid Artery, IMA: Internal Maxillary Artery, MMA: Middle Meningeal Artery, ICA: Internal Carotid Artery.

### Case 1: Intracranial extension to cavernous sinus and infratemporal fossa treated with endoscopic transseptal resection

**Figure 1:**
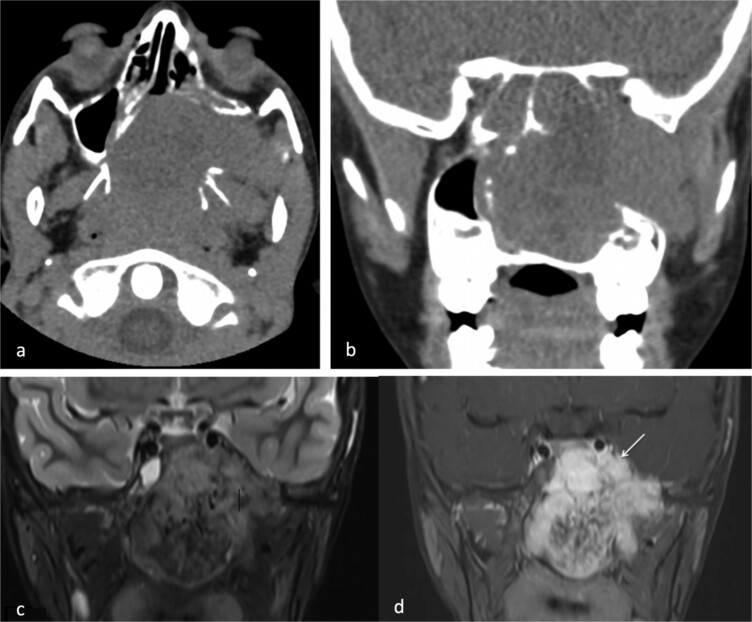
(a) Axial non-contrast CT of the paranasal sinuses showing a soft tissue mass obliterating the posterior aspect of the nasal cavity, with extension and remodeling of the left pterygopalatine fossa into the infratemporal fossa. (b) Coronal image demonstrating superior extension into the left sphenoid sinus and left inferior orbital fissure. (c) Coronal T2-weighted MRI showing a large JNA with heterogeneous signal intensity and multiple flow voids consistent with hypervascularity. (d) Post-contrast coronal T1-weighted MRI demonstrating avid contrast enhancement and invasion of the left Meckel’s cave and cavernous sinus (arrow).

A 9-year-old previously healthy male was referred due to left nasal blockage and mild intermittent epistaxis persisting for 6 months. Nasal endoscopy revealed a clearly evident pinkish mass in the left nasal cavity. Non-contrast Computed Tomography (CT) of the paranasal sinuses (Fig. 1a and b) demonstrated a large, expansile, and ill-defined soft tissue mass with its epicenter in the left nasal cavity and nasopharynx. It extends to the left pterygopalatine fossa and the pterygomaxillary fissure, reaching the masticator space. The mass caused significant bone remodeling and expansion, with some erosions noted at the body and the left greater wing of the sphenoid bone, as well as the medial and lateral pterygoid plates. Additionally, the mass was seen extending to the lateral walls of the maxillary and ethmoid sinuses, bilateral sphenoid sinuses, and the inferior bony orbital wall. Contrast-enhanced Magnetic Resonance Imaging (MRI) showed a predominantly left-sided nasopharyngeal mass measuring 6.9 × 5.9 × 5 cm with increased vascularity (Fig. 1c). The mass replaced the left maxillary antrum, posterior ethmoid air cells, and sphenoid sinuses and invaded the left cavernous sinus (Fig. 1d). A pre-operative left ICA angiogram demonstrated the tumor’s blood supply originating from the vidian artery and inferolateral trunk, with the main supply arising from branches of the left internal maxillary artery (IMA). The vascular branches feeding the tumor from both IMAs were embolized.

**Figure 2:**
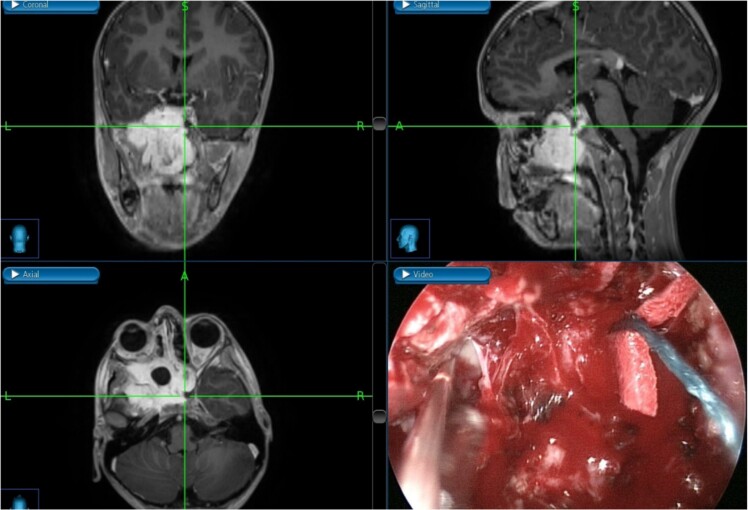
Tumor dissection from the right ICA using intraoperative navigation assistance,with real-time display of instrument location in axial, coronal, and sagittal views.

The patient underwent an endoscopic four-handed transseptal resection of the left JNA following uneventful embolization. Intraoperative endoscopic examination of the nasal cavity revealed a bluish mass in the left nasal cavity and a severe right-sided deviation of the nasal septum. A septal window was created, allowing bilateral access to the tumor. The tumor was dissected and removed, separating it from its bony attachments to the nasopharynx and clivus. Dissection extended to the bilateral sphenoid sinuses, allowing for complete tumor excision from the carotids, cavernous sinuses, infratemporal fossa, and skull base with navigation assistance (Fig. 2). The patient was asymptomatic on close follow-up until 8 months later, when he had intermittent self-limiting episodes of epistaxis without nasal obstruction. Examination revealed bilateral crustations and a small left nasal mass, with no obvious source of bleeding. Confirmation of tumor recurrence was done, and the patient underwent a successful second procedure for tumor resection.

### Case 2: JNA with critical extension to the ICA

**Figure 3:**
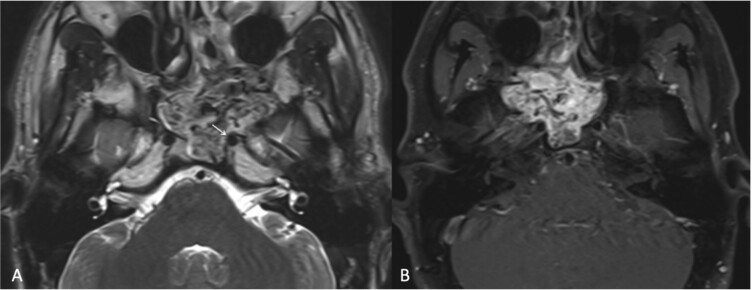
(a) Transaxial T2-weighted MRI image showing a mass with heterogeneous signal intensity and multiple flow voids, along with thinning of the cortical bone overlying the left ICA (arrow). (b) Post-contrast transaxial T1-weighted MRI image demonstrating avid contrast enhancement of the JNA.

A 23-year-old patient presented with left-sided recurrent epistaxis, occipital headache, and nasal obstruction. MRI revealed a hypervascular soft tissue mass measuring 5.1 × 4.5 × 4.7 cm in the posterior aspect of the nasal cavity, with complete obliteration of the left sphenoid sinus (Fig. 3a). The tumor extended laterally into the pterygopalatine fossa, reaching the level of the pterygomaxillary fissure. Post-contrast T1-weighted MRI confirmed marked thinning of the lateral sphenoid wall and abutment of the left ICA (Fig. 3b). The bulk of the blood supply to the tumor was from the left IMA and was successfully embolized.

**Figure 4:**
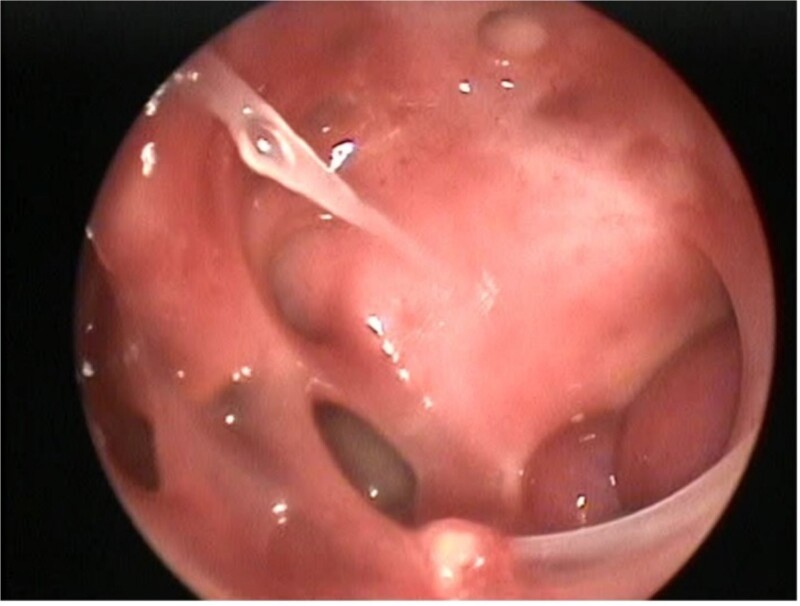
Postoperative nasal endoscopy showing the left ICA within the left lateral sphenoid sinus.

During surgery, the tumor was dissected from the pterygopalatine fossa using bipolar cautery. However, massive bleeding occurred from the tumor pedicle and the maxillary artery, which was controlled with repeated cauterization and packing of the nasal portion of the tumor. The sphenoid sinus was widely exposed by removing the posterior part of the bony septum. The tumor was then dissected from the sphenoid sinus, where further massive bleeding was encountered. This was successfully managed by applying Surgicel to the sphenoid sinus and pterygopalatine fossa. The postoperative endoscopic view of the left ICA within the left lateral sphenoid sinus is shown in Fig. 4.

### Case 3: Resection of a very large tumor with minimal blood loss

A 13-year-old boy had a 5-month history of progressive left-sided nasal obstruction and infrequent mild epistaxis, then noticed a nasal mass protruding from the left lateral nasal wall. On anterior rhinoscopy, a pinkish-brown, smooth, firm, lobular mass was seen filling the left nasal cavity. A CT scan with contrast and brain MRI showed the vascular tumor originating from the pterygopalatine fossa, extending to the nasopharynx. Pre-operative embolization was done to the feeding arteries. Intraoperatively, the mass was found to be attached to the nasal septum near the bony-cartilaginous junction. The tumor base was cauterized using bipolar coagulation. Despite the tumor’s large size (4.0 × 2.5 × 1.0 cm), complete removal was achieved while maintaining a dry surgical field, with an estimated blood loss of 150 cc.

### Case 4: Extreme lateral infratemporal fossa extension

**Figure 5:**
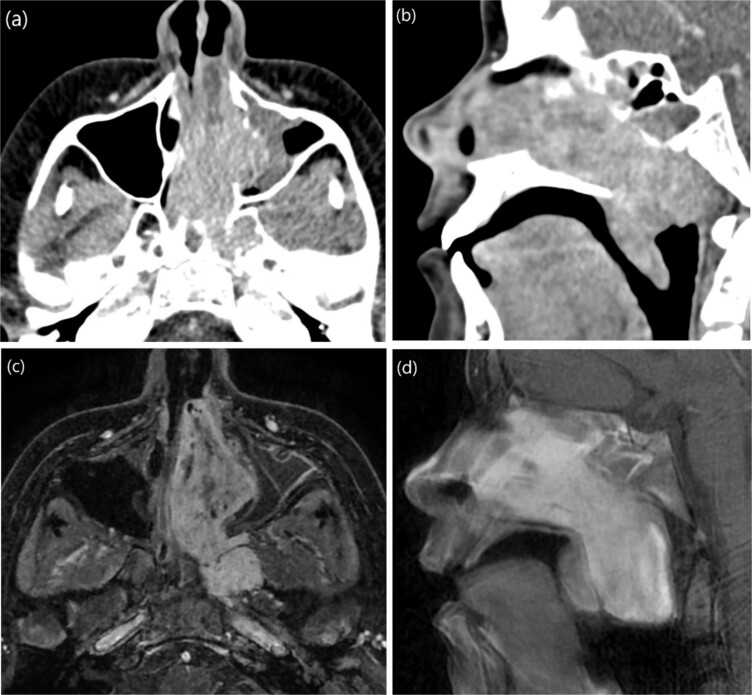
(a, b) Contrast-enhanced CT of the paranasal sinuses in transaxial and sagittal views showing a mass in the left nasal cavity with extension into the nasopharynx. (c, d) Post-contrast T1-weighted MRI in axial and sagittal views illustrating the mass extending laterally into the maxillary antrum and left pterygopalatine fossa, as well as posteroinferiorly into the oropharynx.

A 13-year-old male presented to the hospital with a two-year history of gradually worsening obstructive symptoms. Endoscopic examination revealed a pale, necrotic mass in the left inferior nasal cavity, along with severe septal deviation and bilateral nasal discharge. CT and T1-weighted MRI identified a mass in the left nasal cavity extending posteriorly into the nasopharynx, laterally into the maxillary antrum and left pterygopalatine fossa, and posteroinferiorly into the oropharynx (Fig. 4). The patient underwent preoperative embolization of the branches of the left external carotid artery (ECA), followed by navigation-assisted endoscopic surgical excision (Fig. 5).

**Figure 6:**
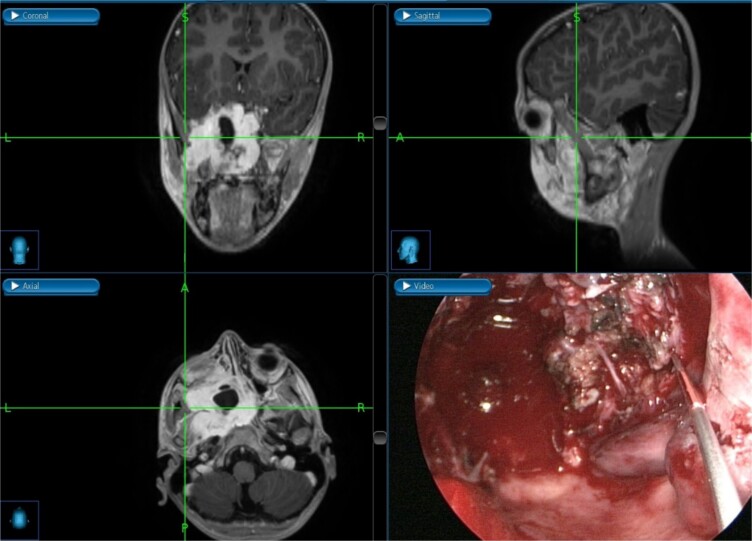
Intraoperative post-excision navigation-assisted images demonstrating the complete removal of the tumor from the far lateral boundary of the infratemporal fossa in axial, coronal, and sagittal views.

The excision was extended laterally to the boundaries of the left infratemporal fossa (Fig. 6). The patient was discharged in stable condition the following day. However, one week later, the patient developed left-sided otalgia, tinnitus, left-sided headache, and clear nasal discharge without any bleeding or obstruction. Five weeks later, he experienced decreased visual acuity in the left eye. Examination revealed a fleshy mass in the pterygopalatine area with purulent discharge, necessitating revision endoscopic excision.

### Case 5: Post-embolization acute cerebrovascular accident

**Figure 7:**
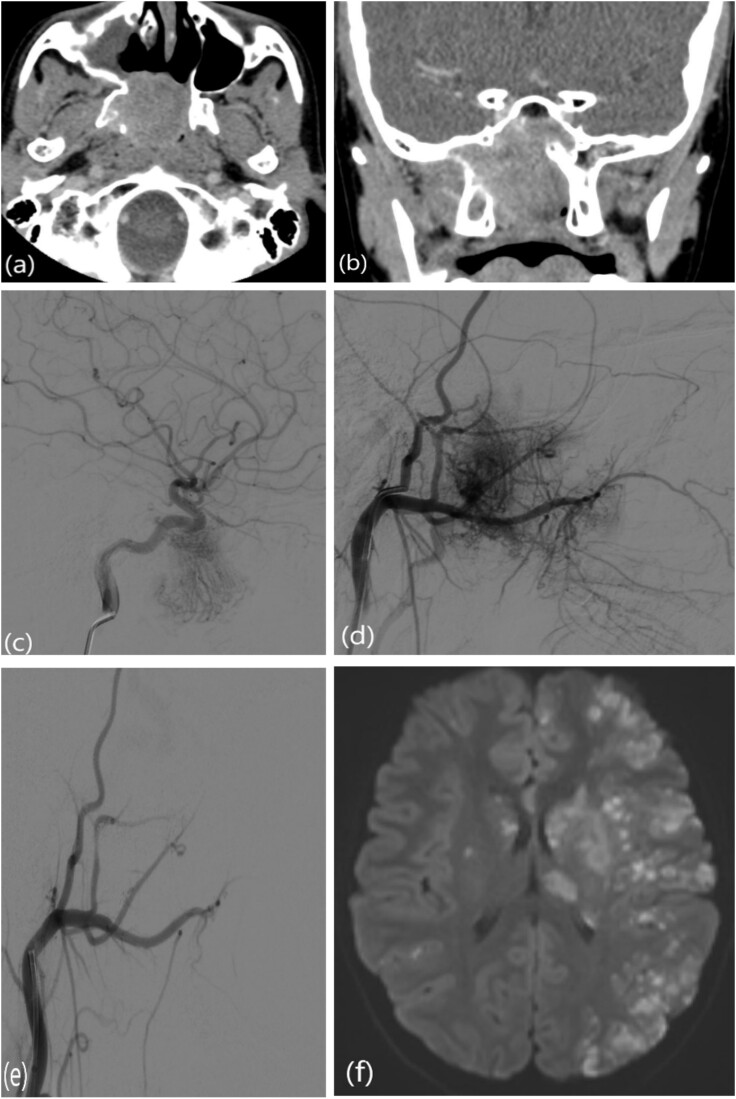
(a, b) Contrast-enhanced CT of the paranasal sinuses showing a right nasopharyngeal mass with extension into the sphenoid sinuses and right pterygoid plate. (c) Lateral projection angiogram of the right ICA demonstrating the blood supply to the right nasopharyngeal mass from the inferolateral trunk. (d) Lateral projection angiogram of the right ECA showing extensive vascular supply to the hypervascular mass from multiple branches. (e) Post-embolization right ECA angiogram showing devascularization of the tumor. (f) Transaxial diffusion-weighted imaging of the brain showing extensive foci of restricted diffusion, predominantly involving the left side, consistent with acute stroke.

**Figure 8:**
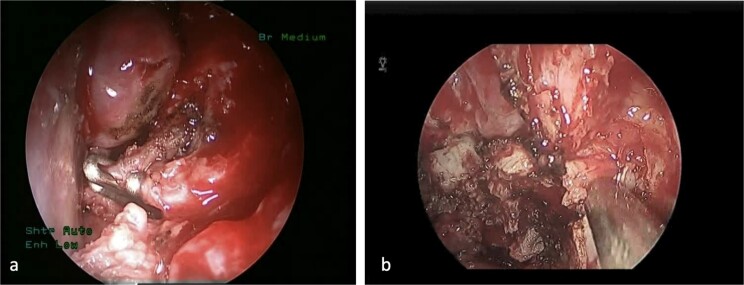
(a) The sphenopalatine artery is clipped before tumor removal. (b) The vascular tumor is dissected from sphenoplataine and pterygopalatine fossa.

This is a case of a 12-year-old male complaining of nasal obstruction and denying epistaxis, headache, and nasal discharge symptoms. He was found to have bilateral JNA and was successfully treated with embolization of the bilateral distal IMA using polyvinyl alcohol (PVA) particles, followed by surgery. A subtotal resection of the nose, involved sinuses, pterygopalatine fossa, and infratemporal fossa was performed with no postoperative complications (Fig. 8). Six months later, the patient had a recurrence. An in-office endoscopic examination revealed a right-sided mass on the posterolateral wall, necessitating resection. One month prior to surgery, the patient was managed in the emergency department for persistent right-sided epistaxis. It lasted for eight hours and was successfully managed with tranexamic acid. CT imaging showed the recurrent right nasopharyngeal mass with skull extension into the sphenoid sinuses and the right pterygoid plate (Fig. 7a and b). Hence, he underwent embolization of the ECA (Fig. 7c–e). The patient subsequently experienced a decreased level of consciousness, aphasia, and right-sided weakness, with a power of 2/5 in both the lower and upper limbs. MRI showed multiple, extensive punctate foci of cortical and basal ganglia acute infarction complicated by edema, predominantly affecting the left side without a hemorrhagic component. Findings are consistent with showering from the PVA particles (Fig. 7f). A total of 12 months later, the patient was recovered and cleared neurologically to undergo endonasal endoscopic removal for the tumor. The patient was treated surgically at another institution, followed by a course of radiotherapy. Thereafter, he resumed his follow-up in our center, showing no evidence of recurrence after 1 year of follow-up.

## Discussion

JNA has a distinctive vascular supply from the ipsilateral ECA, mainly the IMA and ascending pharyngeal artery [2]. Very large lesions can receive additional supply from the contralateral ECA, as seen in Case 1. Moreover, tumor feeders from the ICA are observed in 30% of the cases when the tumor extends to the parapharyngeal space, sphenoid sinus, orbit, or intracranial cavity [3, 11]. Preoperative carotid embolization facilitates resection and reduces the amount of intraoperative bleeding. It is considered the standard of care, except for very small tumors [6, 8, 11, 12]. Although it is associated with serious but rare complications such as cerebral infarcts and blindness, it remains effective [3, 13]. Giorgianni *et al.* managed 79 patients with preoperative embolization using PVC particles and reported effective control of intraoperative bleeding with no hemorrhagic or thromboembolic complications [14].

Endoscopic surgery is associated with minimal risk of hemorrhage, and ligation of feeding branches further reduces blood loss [1, 15]. In fact, the estimated blood loss in open surgeries was higher when preceded by embolization [1]. For pediatric patients, endoscopic approaches help preserve the facial skeleton, which continues to grow until ⁓20 years of age [2, 12]. Additionally, the reported average recurrence rate for purely endoscopic surgeries is 4.7%, significantly lower than the 22.6% observed in open surgeries [1]. One study recorded no long-term sequelae following complete resection over a 10-year period [10]. A literature review by Khattar *et al.* showed that most surgeons opt for open maxillotomy for extensive tumors [5]. JNA with lateral extension to the infratemporal fossa was commonly excised via external craniotomy, especially in cases with intracranial and orbital extension [16]. The infratemporal fossa approach is commonly used for resecting JNA involving the infratemporal fossa, middle cranial fossa, and lateral part of the cavernous sinus [17]. In our institution, we treated JNA extending far laterally into the infratemporal fossa using a navigation-assisted, purely endoscopic approach. Although complete resection in a single procedure was not possible, a revision surgery effectively eradicated the tumor.

The two-surgeon transnasal approach provides a multi-angled view, allowing surgeons to carefully address the relationships between the tumor, middle turbinate, and nasal septum when anterior extension is present. It also provides adequate access to and manipulation of the tumor’s posterolateral portion [18–20]. In the Midilli *et al.* series, 12 patients underwent endoscopic transnasal surgery. The authors advised partial middle turbinate resection to increase the field of view and, in some cases, detach the anterior part of the mass [12]. Additionally, ligation of the sphenopalatine artery at the beginning of the procedure is necessary to allow for minimal bleeding [2]. In a series of 19 patients, Huang *et al.* retracted the tumor through a posterior septal perforation into the contralateral nasal cavity without dividing it intranasally [8]. Recurrence was not reported in any of their cases. Despite the advantages, the “learning curve” and detailed knowledge of endoscopic anatomy remain significant limitations for extensive endoscopic surgeries [9, 18]. Our first case underwent an uneventful resection of JNA using the endoscopic two-surgeon transnasal approach with no postoperative complications and no residual tumor.

## Conclusion

Endoscopic resection was effective and safe in managing JNA extending laterally into the infratemporal fossa, in close proximity to the ICA despite cortical bone thinning, or in cases with extensive bilateral vascular supply. In some cases, the two-surgeon transnasal approach is a promising alternative to open surgeries for complete resection of giant JNA. Preoperative embolization reduces intraoperative bleeding and improves the outcomes of endoscopic surgeries; however, careful detection and management of life-threatening complications are warranted.
